# Career commitment and sense of calling in nursing students: a longitudinal study

**DOI:** 10.3389/fmed.2026.1760650

**Published:** 2026-02-13

**Authors:** Tingting Ding, Jiaqi Shi, Wenjing Li, Feifei Xiong, Huihui Xu, Chengjia Zhao, Guohua Zhang, Chen Cong

**Affiliations:** 1 The First Affiliated Hospital of Wenzhou Medical University, Wenzhou, China; 2 School of Nursing, Wenzhou Medical University, Wenzhou, China; 3 Department of Psychology, School of Mental Health, Wenzhou Medical University, Wenzhou, China; 4 School of Education, Renmin University of China, Beijing, China; 5 Key Research Center of Philosophy and Social Sciences of Zhejiang Province, Institute of Medical Humanities, Wenzhou Medical University, China; 6 The Affiliated Kangning Hospital, Wenzhou Medical University, Wenzhou, China; 7 Department of Publicity Wenzhou Medical University, Wenzhou, China

**Keywords:** career commitment, career self-efficacy, nursing students, only child and non-only child, sense of calling

## Abstract

**Background:**

Under China’s one-child policy, the new generation of nursing students has gradually entered the workforce. Their career commitment, career self-efficacy, and sense of calling have undergone changes. It is necessary to assess the relationships and underlying mechanisms among these factors in order to stabilize the nursing workforce and cultivate high-quality clinical nursing professionals.

**Methods:**

A cross-lagged design was used, with a total of 693 nursing students participating in September and December 2022. The 27-item undergraduate career commitment scale, the Chinese version of career self-efficacy scale, and the brief calling scale were measured over time.

**Results:**

The relationships between career commitment, career self-efficacy, and sense of calling differed by only child and non-only child nursing undergraduates.

**Conclusion:**

It is important to introduce specific measures aimed at enhancing the quality of nursing education and implementing effective nursing management methods to ensure the stability of the nursing workforce.

## 1 Background

As the Chinese population continues to age, the demand for medical care is on the rise. Following the implementation of the one-child policy, the working-age population in China (aged 16–59) decreased by over 40 million, while the proportion of the population aged 65 and above has reached 13.50% (1). However, China has approximately 5 million registered nurses, which remains insufficient to meet the increasing demands of population aging (2). The demographic dividend in China appears to be diminishing. Due to the high risks, intensity, and pressure involved in nursing work, the turnover rate among nurses remains relatively high (3). The extremely imbalanced supply and demand scenario further exacerbates the strain on medical and healthcare resources, underscoring the critical need to foster qualified nursing talent reserves. However, nursing students from the one-child generation appear to exhibit limited enthusiasm for pursuing careers in the nursing industry, and issues of low sense of belonging and a lack of professional identity are also observed (4). Therefore, it is particularly important to study the relationships between professional commitment, professional efficacy, and professional mission among nursing students. This effort aims to enhance their sense of belonging and identity toward the nursing profession, ultimately contributing to the stabilization of the nursing talent reserve.

### 1.1 Career commitment and career self-efficacy

Career commitment refers to the commitment individuals make to their career goals and professional development, which is a set of values about their career (5). Career development theory (6) suggests that individuals’ entry into a particular industry is influenced by various factors, including interests, abilities, values, needs, education, degree of social resource utilization, social occupational structure, and trends. During the phase of acquiring professional knowledge and skills, nursing students’ interest and perception of the profession are crucial to their decision to pursuing nursing (7). Moreover, Career commitment plays an important role in the career development of nursing students. Nursing students who possess a strong career commitment are able to define their career goals and development trajectory, enhancing the effectiveness of their career advancement. This commitment also serves as a driving force, motivating them to consistently pursue growth and attain professional success in their nursing careers (8). Study (9) has also shown a negative correlation between career commitment and intention to leave the profession, further demonstrating the importance of career commitment in the career development of nursing students.

Career self-efficacy refers to an individual’s confidence and belief in their ability to achieve expected goals and deliver commendable performance in their work. This confidence stems from personal experiences, skills, knowledge, and emotions, and serves as a pivotal gauge for individuals to assess their professional competence (10) Previous studies have demonstrated that nursing students with higher career self-efficacy can better cope with professional challenges and difficulties, This, in turn, results in enhanced work efficiency and quality, thereby augmenting their professional competitiveness and potential for career development (10). During the exploration stage (ages 19–24), adolescents engage in a process of discovering their abilities, roles, and potential occupations through school activities, club engagements, part-time jobs, and other opportunities (11). As the preliminary determination and validation of a career as a viable long-term professional path takes shape, it gives rise to the formation of commitment to the profession (career commitment) and confidence in one’s professional capabilities (career self-efficacy). Positive psychology (12) also reveals an intrinsic connection between these two aspects: positive cognition and emotions can play a constructive role in personal wellbeing development. In particular, when nursing students have a strong belief in the nursing profession, it promotes their professional confidence and enhances their sense of happiness (13). Previous cross-sectional studies have shown a positive correlation between career commitment and career self-efficacy (14). Therefore, this study hypothesizes that there is a reciprocal relationship between career commitment and career self-efficacy among nursing students over time (H1).

### 1.2 Career commitment and sense of calling

Due to the expansion of higher education in nursing and dissatisfaction with clinical nursing work, some nursing students may choose not to pursue the nursing profession after graduation. A lower sense of calling can be an internal factor contributing to this decision (15). Sense of calling refers to an individual’s cognition and perception of the importance, significance, and value of their chosen profession. It is an emotional connection between individuals and their profession, serving as an intrinsic driving force (16). Individuals with a strong sense of calling tend to show greater enthusiasm for professional learning, strive for higher standards, and actively pursue career development. Also, sense of calling can help individuals overcome setbacks and difficulties in their work, enhancing their sense of self-worth and self-confidence (17).

Study (18) suggests that nurses with a stronger sense of calling exhibit heightened career commitment, a reduction in negative workplace behaviors, and an enhancement in mental wellbeing. In a study conducted by Afsar et al. (19), a questionnaire survey involving 294 nurses was carried out to explore the relationship between sense of calling and career commitment. The results indicates that sense of calling plays a complete mediator between career commitment, organizational citizenship behavior, workplace deviant behavior, and turnover intention. Based on these premises, this study hypothesizes the existence of a mutually predictive relationship between career commitment and sense of calling among nursing students (H2).

### 1.3 Career self-efficacy and sense of calling

According to the career development theory (20), during the late exploration stage, individuals select a specific occupational field and start experimenting with the feasibility of their career development goals. Brown and Lent (21) indicate that individuals with high career self-efficacy often have more confidence and positivity in achieving their aspirations, thus promoting work motivation. Conversely, the essence of a calling is rooted in individual interests, purpose, and motivation. Positive feedback from nursing students’ explorations of their career’s feasibility can bolster their confidence in their chosen path, thereby reinforcing their commitment and dedication to their duties of their career’s feasibility can bolster their confidence in their chosen path, thereby reinforcing their commitment and dedication to their duties. Zhang et al. (22) demonstrate a positive correlation between career self-efficacy and sense of calling. Similarly, Yang et al. (23) pointed out that nursing students’ career self-efficacy and sense of calling reach a moderate level after significant public health events, and occupational self-efficacy can predict the emergence of sense of calling. Additionally, when individuals perceive that their work has meaning and social impact, they are more inclined to enhance their skills and fully utilize their abilities (24). Consistently achieving success and attaining goals in their profession gradually strengthens individuals’ career self-efficacy (25). Building upon these observations, this study proposes a reciprocal predictive relationship between career self-efficacy and the sense of calling among nursing students over time (H3).

### 1.4 Differences between only child and non-only child nursing students

Over the past three decades of implementing the one-child policy in China, nursing students who have been influenced by it have gradually entered the workforce. Surprisingly, few attention have been paid to nursing students who are once again affected by this policy. In this context, a generation of young people with distinct characteristics has emerged (26). Raised in an environment fostering independent thought and autonomous problem-solving from an early age, they exhibit a strong sense of independence. Moreover, they benefit from diverse family resources and showcase higher intellectual and learning capabilities (27). However, some shortcomings are also evident: occasional unreliability, limited competitiveness, a propensity toward pessimism, and a somewhat subdued sense of responsibility, leading to them being colloquially referred to as the “little emperors” generation (28). The professional cognition and attitudes of this generation of nursing students will have an impact on the stability of the entire nursing team (29). Therefore, it is necessary to assess the relationships between career commitment, career self-efficacy, and sense of calling among nursing students, especially concerning whether they are from one-child households. This study hypothesizes that discernible disparities exist between one-child and non-one-child nursing students in terms of career commitment, career self-efficacy, and the sense of calling, as examined through a cross-lagged model (H4).

### 1.5 The present study

While numerous studies have explored the pairwise relationships between career commitment, career self-efficacy, and sense of calling, there remains a notable research gap concerning the interplay among these three factors. Furthermore, most of the existing studies are cross-sectional nature, limiting their ability to infer causal relationships accurately It is worth noting that China has implemented the one-child policy for over forty years, and the generation of nursing students affected by this policy is becoming the backbone of the nursing field. In this context, there is still a limited amount of research on nursing students. This study aims to elucidate the impacts of the one-child policy on the relationships and underlying mechanisms between career commitment, career self-efficacy and sense of calling among nursing students. The research hypotheses are as follows:

*H1*: There is a reciprocal relationship between career commitment and career self-efficacy among nursing students over time.

*H2*: There is a reciprocal relationship between career commitment and sense of calling among nursing students over time.

*H3*: There is a reciprocal relationship between career self-efficacy and sense of calling among nursing students over time.

*H4*: There are differences in the cross-lagged relationships among career commitment, career self-efficacy, and sense of calling between only-child and non-only-child student groups.

## 2 Materials and methods

### 2.1 Participants and procedure

The initial number of young people available for participation was 744 participants, all participants were third-year undergraduate nursing students from a university in Zhejiang Province, China. Measurements were taken in September 2022 (T1) and December 2022 (T2), which corresponded to a key transition period in the nursing curriculum from pre-clinical learning to initial clinical placement exposure. Consequently, the final sample comprised 693 students (588 females and 105 males).

The sample size was considered adequate for cross-lagged panel modeling, as SEM guidelines (30) commonly recommend a minimum sample size of approximately N ≥ 200 for stable parameter estimation. Therefore, the final valid sample (*N* = 693) was more than sufficient for the proposed analyses.

This study was approved by the Research Ethics Committee at the first author’s institution. Prior to the investigation and data collection, participants were informed of the purpose and procedures of the study. Each participant’s student ID (but not their name) was recorded only for matching the longitudinal data. The researchers also assured the confidentiality of the data, which could only be accessed by authorized researchers. Thus, the data from this study would be anonymous. Self-report questionnaires were distributed to all students through Wenjuanxing, an online crowdsourcing platform in China, during each wave. Participants were provided with information about local professional help resources in case they needed them. The demographic information is presented in Table 1.

**TABLE 1:** Demographic information of the sample.

Demographic information	Level	*N*	Percentage	*M*(*SD*)
Gender	Female	588	84.8	
	Male	105	15.2	
Residence	Urban	281	40.5	
	Rural	412	59.5	
One-child family	No	448	64.6	
	Yes	245	35.4	
Father’s education level	Elementary or lower	117	16.9	
	Junior middle school	320	46.2	
	Senior middle school	174	25.1	
	Bachelor’s degree or higher	82	11.8	
Mother’s education level	Elementary or lower	175	25.3	
	Junior middle school	303	43.7	
	Senior middle school	141	20.3	
	Bachelor’s degree or higher	74	10.7	
Whether they have relatives practicing medicine	Yes	131	18.9	
	No	562	81.1	
Age				20.3(1.2)

### 2.2 Measures

#### 2.2.1 Sociodemographic questionnaire

Demographic details included gender, age, place of origin (whether from a rural or urban area), and presence of other siblings. Responses to questions about educational and vocational status were also collected which included the highest completed level of the parent’s education and whether they had relatives practicing medicine.

#### 2.2.2 Career commitment

The 27-item Undergraduate Career Commitment Scale was used to assess the four dimensions of career commitment among college students: (a) affective commitment, (b) continuance commitment, (c) normative commitment, and (d) ideal commitment (31). Participants were asked to indicate on a five-point scale ranging from 1 = *totally disagree* to 5 = *absolutely agree*. A higher score indicated a stronger commitment to the respective characteristic. Cronbach’s alpha for the two time points were 0.94 and 0.95, respectively.

#### 2.2.3 Career self-efficacy

Career self-efficacy was measured by the Chinese adaptation (32) of the career-related self-efficacy scale (33). This scale comprises two items, which are divided into two dimensions: educational requirements (e.g., “How confident you are to complete the education or training required of your career successfully”) and job responsibilities (e.g., “How confident you are to complete the duties of your career successfully”). Responses were rated on a scale ranging from 1 (have no confidence) to 5 (very confident). A higher score indicated greater career self-efficacy. Cronbach’s alpha for the two time points were 0.88 and 0.91, respectively.

#### 2.2.4 Sense of calling

The presence of sense of calling was tested using the Brief Calling scale developed by Steger et al. (34). The scale consists of two items (e.g., “I have a calling to a particular kind of work.” and “I have a good understanding of my calling as it applies to my career.”) rated on a scale ranging from 1 (not at all true of me) to 5 (totally true of me). A higher score indicated a stronger sense of calling for the respective characteristic. The scale has been demonstrated to be reliable and valid for assessing calling in the Chinese cultural context (35) Cronbach’s alpha for the two time points were 0.91 and 0.93, respectively.

### 2.3 Data analysis

Descriptive statistics, correlations, independent *t*-tests, and repeated measures analysis of variance were conducted using SPSS 24.0. To examine the longitudinal associations among career commitment, career self-efficacy, and sense of calling, cross-lagged panel models were estimated. Multi-group cross-lagged models were further conducted to compare path estimates between only-child and non-only-child students. Model interpretation emphasized standardized coefficients and confidence intervals, with attention to the overall pattern and consistency of effects across models. All models were tested via maximum likelihood (ML) estimation in Mplus 8.3 to estimate the structural equation model. Initially, we tested the configural, metric, and scalar invariance of measurement models by imposing equality constraints to ensure that the latent constructs had a comparable measurement structure across time. Subsequently, we conducted a two-wave cross-lagged model, including auto-regressions and regression paths, to examine the relationships among career commitment, career self-efficacy and sense of calling.

To evaluate the model fit, the following criteria (36) were used: the chi-square value divided by the degrees of freedom (χ^2^/*df*) between 2 and 3, the root mean squared error of approximation (RMSEA) lower than 0.08, the standardized root mean square residual (SRMR) less than 0.08, the Tucker and Lewis Index (TLI) and the comparative fit index (CFI) values greater than 0.90 were considered as indicators of an acceptable model fit. Additionally, cognizing that χ^2^ is sensitive to sample size and frequently yields statistically significant values, the current study used ΔCFI, ΔTLI, and ΔRMSEA to compare measurement models. The ΔCFI and ΔTLI values were both less than 0.010, and ΔRMSEA values < 0.015, indicating that the fit between the models was equivalent.

## 3 Results

### 3.1 Descriptive statistics and difference test

In Table 2, means, standard deviations, and correlations for the measures of career commitment, career self-efficacy and calling are shown. The skewness and kurtosis of these variables fell within the acceptable range. Furthermore, all study variables were significant and positive in each wave. Then, the differences in career commitment, career self-efficacy, and sense of calling at T1 and T2 between participants with and without siblings were tested. Interestingly, the career commitment of only-child students was higher than that of non-only-child participants at both T1 and T2, as shown in Table 3. However, the pairwise comparisons revealed no significant differences in career commitment, career self-efficacy, and sense of calling between T1 and T2.

**TABLE 2:** Descriptive statistics and correlations among variables of interest.

Variables	1	2	3	4	5	6
T1 career commitment	1	1	1	1	1	1
T1 career self-efficacy	0.50***					
T1 calling	0.55***	0.59***				
T2 career commitment	0.57***	0.39***	0.47***			
T2 career self-efficacy	0.41***	0.50***	0.45***	0.53***		
T2 calling	0.45***	0.43***	0.53***	0.55***	0.66***	
M	94.80	15.31	7.76	95.47	15.47	7.86
SD	14.19	3.07	1.68	14.82	3.21	1.67
Skewness	−0.31	−0.63	−0.44	−0.26	−0.76	−0.50
Kurtosis	1.25	0.89	−0.09	1.43	1.07	0.06

***p < 0.001, the same below.

**TABLE 3:** Differences between only-child and non-only-child among research variables.

Variables	Only or non-only-child M (SD)		*t*
	Only-child *(N* = 245)	Non-only-child (*N* = 448)	
T1 career commitment	96.64(15.77)	93.79(13.15)	−2.54**
T1 career self-efficacy	15.57(3.13)	15.16(3.03)	−1.67
T1 sense of calling	7.90(1.70)	7.68(1.67)	−1.61
T2 career commitment	97.25(14.25)	94.49(15.05)	−2.35**
T2 career self-efficacy	15.58(3.38)	15.42(3.12)	−0.63
T2 sense of calling	7.89(1.78)	7.84(1.61)	−0.32

**p < 0.01.

### 3.2 Cross-lagged model testing

**FIGURE 1:**
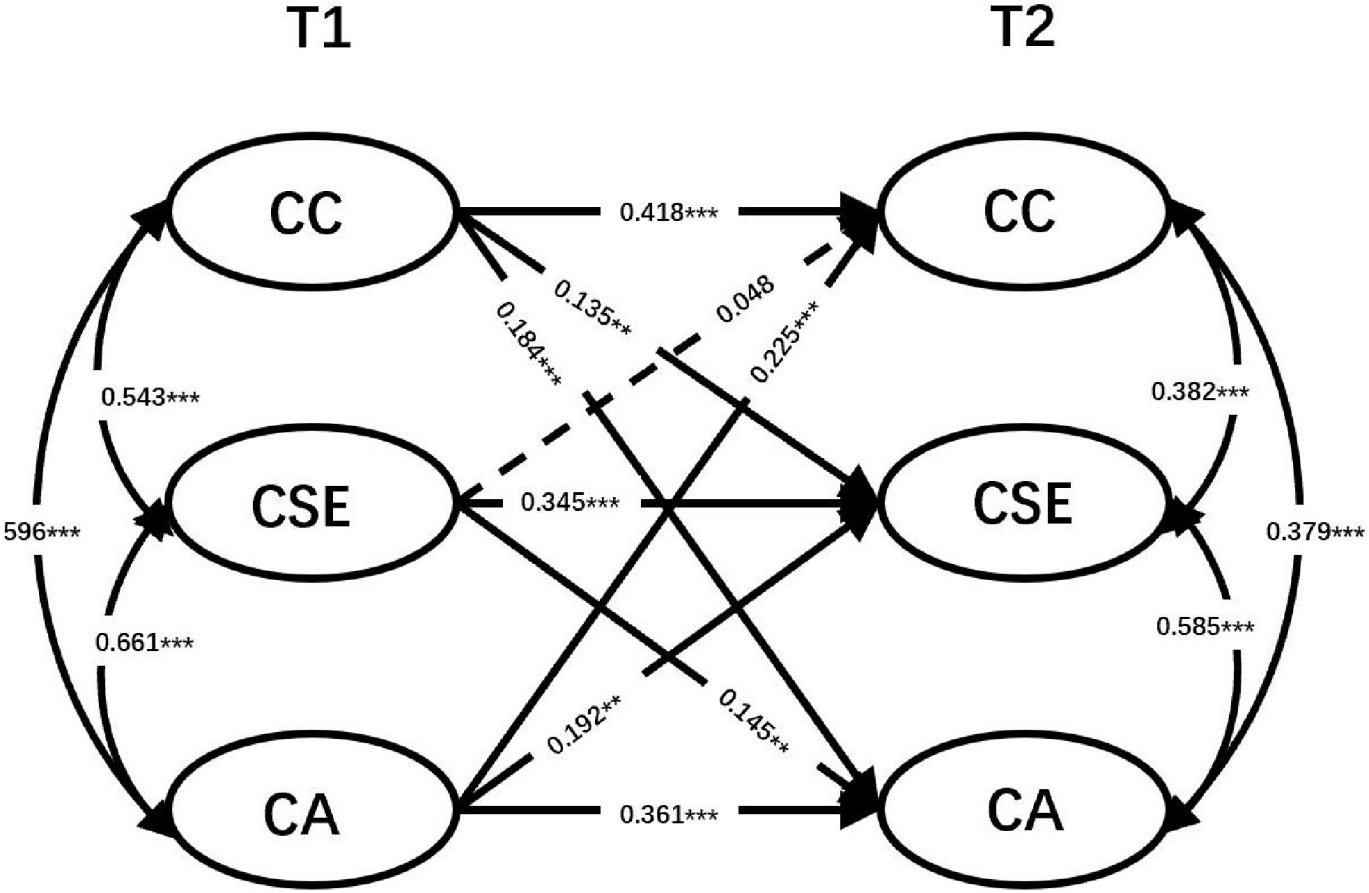
A cross-lagged model for all subjects. CC, career commitment; CSE, career self-efficacy; CA, sense of calling. Dashed lines indicate non-significant estimates. All the reported parameters are standardized. Coefficients represent standardized values. *N* = 693, the same below.

A cross-lagged panel model was analyzed to test the longitudinal and bidirectional relationships among the career commitment, career self-efficacy and sense of calling. To control for cross-sectional correlations, correlations among the three variables at the same measurement points were included. The results revealed significant within-time correlations between career commitment, career self-efficacy, and sense of calling at both measurement points within the cross-lagged panel model. The model exhibited a good fit to the data (χ^2^ = 216.577, *df* = 81, p < 0.001; CFI = 0.987, TLI = 0.981, RMSEA = 0.049 [0.041, 0.057], SRMR = 0.020). As illustrated in Figure 1, autoregressive paths were significant for the career commitment, career self-efficacy and calling across time. In terms of cross-lagged effects, it was observed that higher levels of career commitment at T1 positively predicted higher levels of calling at T2 (β = 0.184, *p* < 0.005), and vice versa, higher levels of calling at T1 positively predicted higher levels of career commitment at T2 (β = 0.225, *p* < 0.001). Furthermore, higher levels of career commitment at T1 predicted higher levels of career self-efficacy at T2 (β = 0.135, *p* < 0.01), while the reverse relationship (career self-efficacy at T1 predicting career commitment at T2) was not significant (β = 0.048, *p* > 0.05). As expected, the association between career self-efficacy and calling was bidirectional: career self-efficacy at T1 positively predicted calling at T2 (β = 0.145, *p* < 0.01), and also calling at T1 positively predicted career self-efficacy at T2 (β = 0.192, *p* < 0.01) (see Table 4).

**TABLE 4:** Structural paths result of all latent variables.

Path	Estimate (β)	SE	90% CI (Lower)	90% CI (Upper)	*p*-value
CC (T1) → CC (T2)	0.418	0.04	0.34	0.496	***
CSE (T1) → CSE (T2)	0.345	0.038	0.271	0.419	***
CA (T1) → CA (T2)	0.361	0.042	0.279	0.443	***
CC (T1) → CSE (T2)	0.135	0.045	0.047	0.223	**
CC (T1) → CA (T2)	0.184	0.049	0.088	0.28	***
CSE (T1) → CC (T2)	0.048	0.04	−0.03	0.126	0.23
CSE (T1) → CA (T2)	0.145	0.05	0.047	0.243	**
CA (T1) → CC (T2)	0.225	0.046	0.135	0.315	***
CA (T1) → CSE (T2)	0.192	0.052	0.09	0.294	***

**p < 0.01,

***p < 0.001.

### 3.3 A comparison of differences between only-child and non-only-child students

**FIGURE 2:**
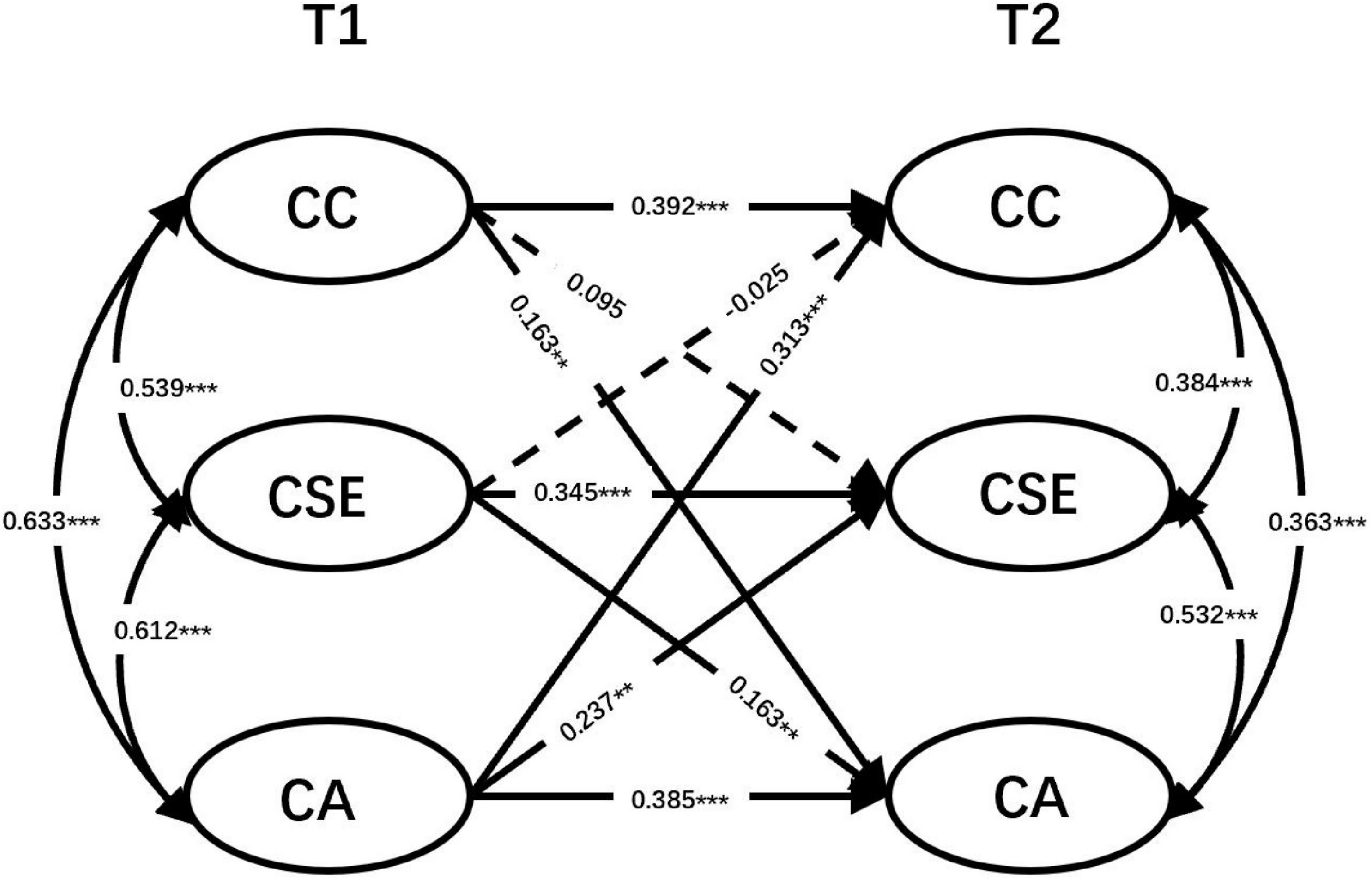
Cross-lagged panel model (non-only-child group). Same conventions as Figure 1. All coefficients are standardized (β). *N* = 448.

**FIGURE 3:**
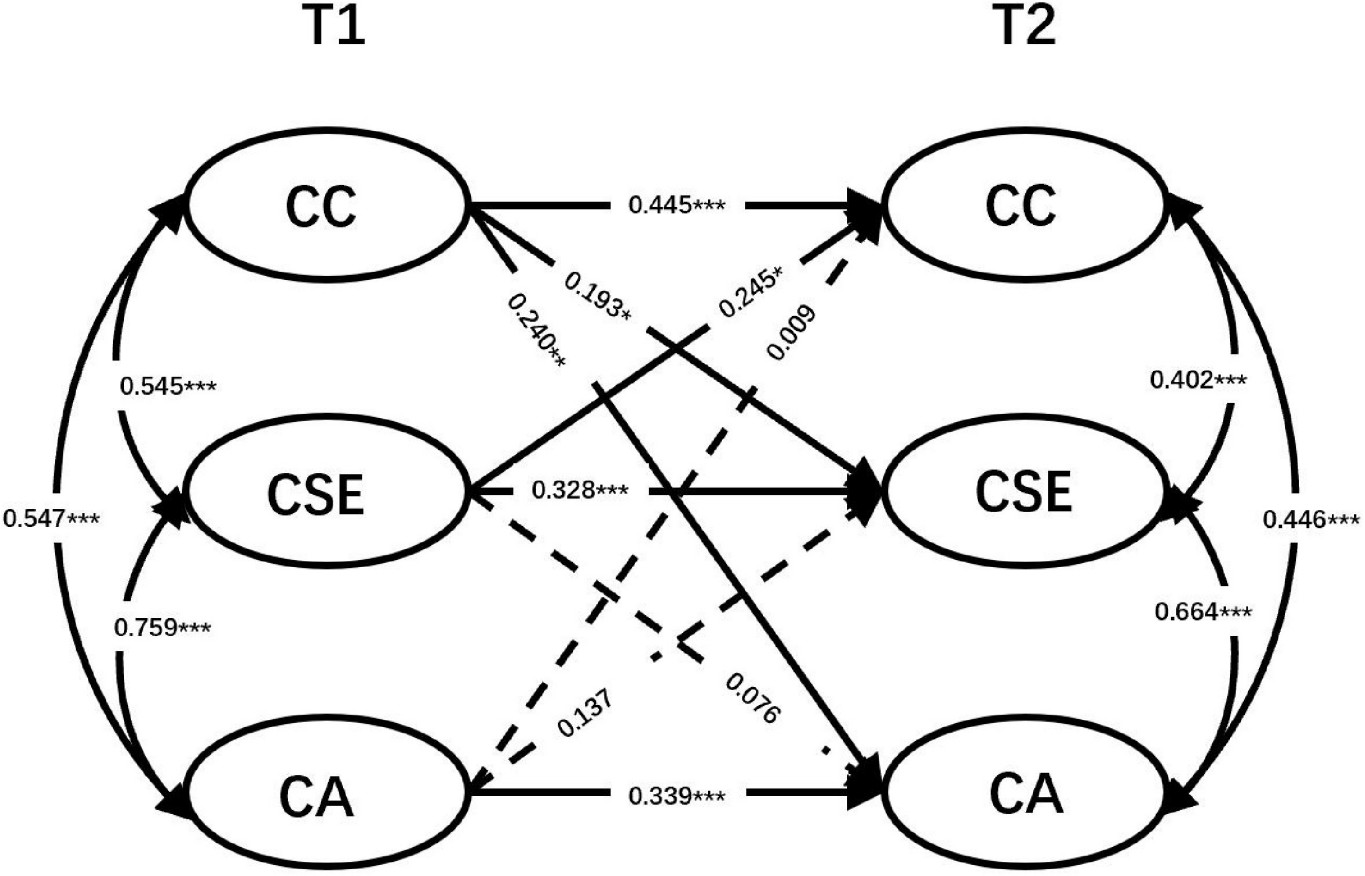
Cross-lagged panel model (only-child group). Same conventions as Figure 1. All coefficients are standardized (β). *N* = 245.

Multi-group comparative structural equation modeling was initially conducted to examine whether the career commitment, career self-efficacy and sense of calling were measured the same for both only-child and non-only-child students over time (see Table 5). As shown in Table 4, the ΔCFI and ΔTLI values < 0.010, respectively, and ΔRMSEA values < 0.015. Therefore, the configural, metric, and scalar invariance of latent constructs were all established. We first tested configural, metric, and scalar invariance across groups. Configural invariance allowed parameters to vary freely across groups. Metric and scalar invariance were then examined by constraining factor loadings and item intercepts to be equal across groups., and the model demonstrated an acceptable fit to the data [χ^2^(204) = 393.51, *p* < 0.001; CFI = 0.98; TLI = 0.98; RMSEA = 0.05; SRMR = 0.07]. A fully unconstrained measurement model was then analyzed in which all parameters constrained to be the same for both groups, χ^2^(198) = 382.91, *p* < 0.001, CFI = 0.98, TLI = 0.98, RMSEA = 0.05, SRMR = 0.06. The chi-squared test of difference indicated that the fully unconstrained measurement model fit the data equally well compared to the unconstrained model [Δχ^2^(6) = 10.60, *p* > 0.05]. Thus, the unconstrained model was then used in subsequent analysis. In contrast to the general model, it was observed that in the non-only-child group, career commitment at T1 did not predict career self-efficacy at T2 (β = 0.095, *p* > 0.05), and career self-efficacy at T1 also did not predict career commitment at T2 (β = −0.025, *p* > 0.05). However, in the only-child group, it was found that career commitment and career self-efficacy positively predicted each other (see Figures 2, 3).

**TABLE 5:** Measurement model tests of all latent variables.

Variables	χ*2/df*	CFI	TLI	RMSEA (90% CI)	SRMR	ΔCFI	ΔTLI	ΔRMSEA
Configural invariance	2.67	0.987	0.981	0.049([0.041, 0.057])	0.020			
Metric invariance	2.56	0.987	0.982	0.048([0.040, 0.055])	0.023	< 0.001	0.001	0.001
Scalar invariance	2.54	0.987	0.983	0.047([0.040, 0.055])	0.022	< 0.001	0.001	0.001

## 4 Discussion

Based on Holland (1959)’s career development theory (37), this study constructed a cross-lagged model of career commitment, career self-efficacy, and sense of calling among Chinese nursing undergraduates. Additionally, it compared the differences between students who are only children and those who are not. This holds both theoretical and practical significance in terms of sustaining a stable cohort of nursing professionals from the new generation and enhancing the psychological wellbeing of emerging nursing professionals.

### 4.1 The cross-lagged relationships between career commitment, career self-efficacy, and sense of calling among nursing undergraduates

In this study, we found that career commitment at T1 significantly predicted career self-efficacy at T2, partially supporting H1. This finding aligns with previous research (10). From the perspective of positive psychology nursing students’ career commitment fosters a sense of identification and belonging to the nursing profession. These constructive cognitions accelerate their development and boost their confidence in career growth. For example, ceremonies like receiving the nursing cap and taking an oath enhance emotional attachment among nursing students, forming the basis for their future professional journey. However, contrary to the current research findings, career self-efficacy at T1 did not predict career commitment at T2 (38). This difference can be attributed to nursing students in the professional exploration phase (6), as they need to transition between the roles of a student and a nurse. During this phase, they face some obvious role conflicts: limited clinical experience, limited communication skills, and unfamiliarity with standardized nursing procedures. This hampers the development of robust career self-efficacy, consequently impeding the further enhancement of career commitment. Consequently, vocational educators should emphasize nurturing nursing students’ enthusiasm and positive perceptions of the nursing profession. To strengthen CSE in a more actionable way, educational approaches should prioritize mastery experiences, vicarious learning, and feedback-based performance improvemen. In nursing education, simulation-based learning and scenario-based practice offer structured opportunities for skills rehearsal and debriefing, and have been associated with improvements in self-efficacy and learning outcomes (39). Importantly, because the reverse pathway was not statistically supported in our model, capacity-building programs may be most effective when paired with strategies that reinforce professional meaning and identity formation, rather than relying solely on competence development to strengthen career commitment.

The research results indicate that there is a mutual-promoting relationship between career commitment and sense of calling, thus providing support for H2. This result is consistent with current research findings (17) and the career development theory (40) that states the more individuals invest in a certain role at a stage, the higher their likelihood of success. Career commitment serves as the emotional investment nursing students make in their professional paths. When nursing students have a higher level of career commitment, they are more likely to achieve greater professional success and subsequently foster the development of a sense of calling (41). Likewise, perceiving the profound significance of the nursing profession intensifies their commitment to it. Therefore, nursing educators should assist nursing students in comprehending the nobility and sanctity associated with the nursing profession. In practice, nurse leaders can strengthen nurses’ sense of calling by using structured reflection and narrative sharing around meaningful patient-care experiences. Reflection can focus on nurses’ value-based decisions, patient-centered interactions, and moments of professional growth, which helps connect everyday tasks with professional purpose and supports more stable career commitment (42, 43).

Our research findings demonstrate that career self-efficacy and sense of calling of nursing students can predict each other over time; H3 was supported. This is consistent with current research results (44). High career self-efficacy among nursing students is manifested by their ability to control the pace of clinical work, make optimal use of clinical resources, and maintain positive expectations for their profession (22). In practice, training programs could be designed to combine meaning-centered components with competence-focused practice. For example, simulation scenarios can be framed around patient-centered ethical dilemmas or family communication challenges, followed by debriefing that explicitly links “clinical competence” with “professional purpose,” thereby strengthening both efficacy beliefs and perceived meaningfulness of nursing work. Simulation-based education is consistently described as beneficial for self-efficacy and learning outcomes, especially when paired with reflection and debriefing (45).

### 4.2 Differences in the cross-lagged model among only-child and non-only child undergraduates

The research findings indicate that there are no differences in the cross-lagged relationships between career commitment and sense of calling among only child. The association between career commitment and sense of calling is not influenced by family structure, but rather relates to individuals’ attitudes and values towards their chosen profession (16). Nursing students, influenced by humanistic nursing education, develop a deep love and sense of responsibility towards the nursing profession. They consider it their mission to serve patients and have a strong desire to listen to, care for, and assist patients (46). If pathways differ between only-child and non-only-child nurses, support should be tailored. Practical options include mentorship matching, peer support, and guided reflection to strengthen meaning at work and coping resources, which may support more stable career orientation (47).

Hypothesis 4 was partially supported, indicating that career commitment and career self-efficacy cannot predict each other in the non-only child group, while they can predict each other in the only child group. With relatively fewer siblings, only child nursing students may find it easier to obtain attention and support from their families and society, which may further strengthen their awareness of professional commitment (48). In contrast, non-only child nursing students, who likely compete for resources within the family, may exert more effort to gain support and attention, stimulating a stronger sense of career commitment (49). Interestingly, career self-efficacy and sense of calling exhibit a predictive relationship among non-only child nursing students but not among only child nursing students. The lack of same-generation companionship in the family environment may be a primary factor contributing to this difference. Research (50) suggests that individuals with siblings tend to be more motivated, work harder, display greater obedience, possess better social skills, and exhibit greater psychological stability compared to only children. The relatively limited competition and responsibility sharing experienced by only children may contribute to their lack of vocational calling (51). Non-only children may need to share family resources and responsibilities, motivating them to pursue their goals and mission more vigorously, thus developing a stronger sense of vocational calling. This highlights the importance for nursing educators to differentiate their approach and provide targeted guidance based on nursing students’ different family structures.

## 5 Limitations and future directions

This study has the following limitations: Firstly, the research sample is relatively narrow, consisting only of nursing undergraduate students from a specific region. Generalizing the findings may require broader samples, and future studies could consider conducting multicenter research. Secondly, this study focused exclusively on nursing undergraduates. Future research could encompass a wider range of educational levels and professions. Thirdly, although a two-wave longitudinal design was adopted, the time interval between measurements was relatively short (three months). While this period corresponded to a key transition stage around the initiation of clinical placement, longer follow-up periods and additional measurement waves are needed to examine long-term developmental trajectories and stability of these constructs. Lastly, although this study is longitudinal, it has limited causal inference capacity compared to intervention studies. Future research could incorporate more intervention designs.

Despite these limitations, the current research has significant implications. This study conducted a longitudinal survey on career commitment, career self-efficacy, and calling among Chinese nursing undergraduates, further enriching the knowledge in the field of nursing Undergraduates’ professional psychology. It also highlights the importance of understanding the perspectives of nursing undergraduates who grew up under China’s one-child policy. From a practical standpoint promoting nursing students’ passion for the profession, ensuring the stability of the future nursing workforce, and fostering the holistic development of nursing students’ physical and mental health are crucial concerns for nursing educators. This study provides intervention targets for nursing educators in addressing these critical issues. The gradual implementation of China’s two-child and three-child policies, along with the evolving identity associated with being an only child, will also influence their perception of the nursing profession. This shift calls for further research and discussion in the future.

## 6 Conclusion

This study draws the following conclusions: Career commitment at T1 significantly predicts career self-efficacy at T2; There is a mutually reinforcing relationship between career commitment and sense of calling, as well as career self-efficacy and sense of calling over time; There are differences in the relationships between career commitment, career self-efficacy, and sense of calling among only child and non-only child. Our research underscores the importance of nurturing professional identity among nursing students for their success in the nursing field. It is essential to pay attention to the career mental health of nursing undergraduates who grew up under China’s one-child policy.

## Data Availability

The raw data supporting the conclusions of this article will be made available by the authors, without undue reservation.
